# Probabilistic detection of volcanic ash using a Bayesian approach

**DOI:** 10.1002/2013JD021077

**Published:** 2014-03-03

**Authors:** Shona Mackie, Matthew Watson

**Affiliations:** School of Earth Sciences, University of BristolBristol, UK

## Abstract

Airborne volcanic ash can pose a hazard to aviation, agriculture, and both human and animal health. It is therefore important that ash clouds are monitored both day and night, even when they travel far from their source. Infrared satellite data provide perhaps the only means of doing this, and since the hugely expensive ash crisis that followed the 2010 Eyjafjalljökull eruption, much research has been carried out into techniques for discriminating ash in such data and for deriving key properties. Such techniques are generally specific to data from particular sensors, and most approaches result in a binary classification of pixels into “ash” and “ash free” classes with no indication of the classification certainty for individual pixels. Furthermore, almost all operational methods rely on expert-set thresholds to determine what constitutes “ash” and can therefore be criticized for being subjective and dependent on expertise that may not remain with an institution. Very few existing methods exploit available contemporaneous atmospheric data to inform the detection, despite the sensitivity of most techniques to atmospheric parameters. The Bayesian method proposed here does exploit such data and gives a probabilistic, physically based classification. We provide an example of the method's implementation for a scene containing both land and sea observations, and a large area of desert dust (often misidentified as ash by other methods). The technique has already been successfully applied to other detection problems in remote sensing, and this work shows that it will be a useful and effective tool for ash detection.

**Key Points:**

1. Presentation of a probabilistic volcanic ash detection scheme
2. Method for calculation of probability density function for ash observations
3. Demonstration of a remote sensing technique for monitoring volcanic ash hazards

## 1. Introduction

Volcanic ash can pose a severe hazard to aviation [*Casadevall*, 1994] and can result in heavy financial losses as areas of airspace are closed or restricted to reduce the risk of encounter. Ash from the 2010 eruption of Eyjafjallajökull in Iceland was carried south into congested European airspace, creating widespread disruption with an economic cost to the airline industry exceeding $1 billion USD [*IATA*, 2010]. Fine ash, transported far from the volcanic source, has also been known to cause problems for human health and agriculture [see, for example, *Horwell*, 2007; *Wilson et al*., 2011]. Effective monitoring and forecasting of any potential ash hazard relies on timely detection of the ash. The large distances that ash can travel and the long periods of time for which it can remain in the atmosphere make satellite-borne sensors important for such monitoring. In addition to their spatial and temporal coverage, satellite-borne sensors have the advantage of already being in place when an eruption occurs, providing near-real-time observations for interpretation. Infrared (IR) sensors are particularly useful as they provide continuous observations day and night, and it is these observations that we focus on here.

Traditional techniques for ash detection in IR satellite imagery are based upon the absorption feature of volcanic ash evident at wavelengths around 10 µm. Ice clouds are known to absorb preferentially toward 12 µm, making the difference between the signal recorded at the two wavelengths a reasonable discriminator between ice and ash clouds [*Prata*, 1989]. This is known as the Brightness Temperature Difference (BTD) method. There are, however, situations when the BTD approach ceases to be effective. For example, temperature inversions and clear skies over desert can produce a very similar signal to that expected for ash and so can confuse the detection [*Simpson et al*., 2000]. The presence of water vapor can create a signal opposite to that produced by ash and so effectively “cancel out” an ash signal [*Prata*, 1989; *Simpson et al*., 2000]. A further criticism of the BTD approach is its dependence on thresholds for the difference between the two signals. These thresholds are generally set by experts experienced in ash detection and can therefore create problems for the technique's implementation if the original expertise is lost from an institution. Inconsistencies can occur if different institutions, with different experts, employ the same technique, as sometimes happens when ash travels from the geographical jurisdiction of one Volcanic Ash Advisory Center (VAAC) into that of another. Moreover, setting thresholds in this way is likely to bias techniques toward effective detection of ash similar to that with which the expert has most experience. More recently, *Clarisse et al*. [2010] have proposed a new technique exploiting hyperspectral observations, whereby the shape of an observed spectrum is compared to a set of reference spectra measured from ash observations. The resulting correlations are then processed to determine whether the observed spectrum is likely to be an ash observation. This approach also relies on thresholds and so is also subject to the criticisms above. Furthermore, it has not yet been widely tested and there is likely to be some sensitivity to the choice of reference spectra. Following from this method, a new sophisticated technique has been proposed, using the mean and covariance of a large number of ash-free spectra and of a large number of ash spectra to find the Mahalonobis distance [*Rencher*, 2002] of an observation from each of these two classes [*Clarisse et al*., 2013]. This is a promising technique with a sound physical basis, but as with the aforementioned approaches, it relies on predefined thresholds. An alternative method has been developed based on effective ash emissivities calculated from observations made at different wavelengths. The ratio of the effective emissivities can be shown to indicate whether or not the observations correspond to ash; see *Pavolonis and Sieglaff* [2010] and *Pavolonis* [2010] for a full description of this method. The effectiveness of this approach should be more independent of atmospheric variability than the BTD approach since the atmosphere corresponding to the observation is accounted for in calculation of the effective emissivities. The problem of selecting reference observations to be representative of all volcanic ash observations is also avoided, but implementation of the technique relies on thresholds again being set by expert judgment. In addition to the above criticisms, these methods all produce a binary result. Volcanic ash is more straightforward to detect in some cases than in others, regardless of the detection technique, since it exhibits a stronger spectral signature in some cases than in others. Ambiguous pixels will always exist, i.e., those that cannot be confidently determined to be ash free or ash contaminated, and it is important to identify such pixels for further analysis. It is inappropriate for any classification to force all observations into either an “ash” or “ash free” class if in reality they sit on the class border (as defined by whatever criteria are used for the discrimination). This is true for any image classification scheme, but it is particularly pertinent to volcanic ash for two reasons. First, “ash” is a very broad class, including different sizes and shapes of particles, comprised of different materials, and the class borders are therefore likely to be blurred, meaning that the number of observations classified with low certainty is likely to be higher than for some other classifications. Second, and perhaps most importantly, hazard monitoring often relies on detected ash in satellite imagery and so it is worthwhile knowing the certainty associated with the detection, and how it varies across the image, when making decisions about safety and economic cost.

### 1.1. The Bayesian Technique

The technique presented here builds on a method already successfully applied to problems of cloud detection [*Merchant et al*., 2005; *Mackie et al*., 2010a, 2010b] and for Saharan dust [*Mackie*, 2009]. It has several significant strengths. Most methods require a cloud detection scheme to be implemented to remove meteorological clouds prior to ash detection; however, this necessarily biases the detected ash toward ash that appears spectrally unlike meteorological cloud. Here clouds and ash are considered simultaneously and so this bias is reduced. The Bayesian technique gives a probabilistic result, whereby ambiguously classified pixels, which cannot be confidently assigned to any class on the basis of the available information, are easily identified. In a hazard monitoring situation, these pixels could be interpreted manually or could be classified according to observations containing different data. The method is computationally efficient, does not require thresholds to be set by expert judgment, and could, in principle, be applied to data from any satellite-borne sensor observing at IR wavelengths for which a radiative transfer model is available, thereby providing a consistent product for different data streams. It exploits time- and space-specific physical information for the imaged scene in a similar way to the Pavolonis technique, characterizing observations of cloud and clear sky according to atmospheric parameters anticipated specifically for the imaged atmosphere. Using these scene-specific definitions to distinguish between cloud, clear, and ash observations should significantly reduce the effect of atmospheric variability on the accuracy of the detection. We describe the methodology behind the technique and present an example of its implementation. This demonstration exploits the available information pertinent to a particular sensor and to a particular imaged scene; however, the framework presented could, in theory, be used to exploit prior information from other sources or to interpret data collected by different sensors.

## 2. Image Data

The Infrared Atmospheric Sounding Interferometer (IASI) instrument sits on board the polar-orbiting Met-Op platforms and so has good coverage of higher latitude areas such as Iceland, which is likely to be the source of any future volcanic ash disruption to UK airspace. It has the advantage of also being suitable for retrievals of SO_2_, which it is often useful to observe simultaneously with volcanic ash [*Carboni et al*., 2012]. IASI records at 8461 spectral channels and has a spatial resolution of approximately 12 km. Hyperspectral techniques to explore the wealth of information contained in each IASI pixel can be computationally expensive, and the method presented here does not exploit this feature of the data. Instead, the technique is conceived as being a useful means by which to limit the volume of data considered by more complex algorithms, which can then be run on identified ash pixels to determine the properties of the observed ash. The only spectral channels used are 10.65 µm, 12.00 µm and, for pixels with solar zenith angle less than 90°, 3.90 µm. These wavelengths are similar to those typically exploited for discrimination between clouds and clear sky [*Saunders and Kriebel*, 1988; *Mackie et al*., 2010a, 2010b], and for discrimination between volcanic ash and clear sky [*Prata*, 1989] in IR data, and are not associated with water vapor absorption bands. It is therefore anticipated that these channels will be effective in discriminating between ash, clouds, and clear sky.

Imagery from the eruption of Eyjafjallajökull was considered appropriate to demonstrate the Bayesian technique because it represents a volcanic incident that impacted heavily on European economies, and the plume was widely observed using a range of instruments, meaning that these results are open to scrutiny. This demonstration considers data acquired soon after the onset of the second explosive phase of the eruption. The image used was acquired on 6 May 2010 on the Met-Op B orbit that began at 20:14 UTC and ended at 21:56 UTC. The results for the selected scene are typical of those calculated for other scenes from this eruption; this particular scene was chosen to demonstrate the technique because it includes observations over both land and sea and includes an area over the Saharan desert (desert dust is often misclassified as volcanic ash due to its similar spectral properties [*Simpson et al*., 2000; *Prata et al*., 2001]). It was considered appropriate to use a night image because the absence of solar radiation-dependent data streams means that ash products derived from IR satellite data are likely to be more heavily relied upon at night. A major problem with the validation of ash detection techniques is the lack of a “true” result against which to assess performance. False color composite images calculated from data collected by the SEVIRI sensor can provide a good qualitative indication of the presence of volcanic ash [*Millington et al*., 2012]. EUMETSAT produce a “dust” and an “ash” image from SEVIRI data using channel combinations specifically chosen so that ash and dust appear pink (documentation for these products is provided online: http://oisww.eumetsat.org/IPPS/html/MSG/RGB). These products, from data acquired at 20:00 and at 21:00 on 6 May 2010, are shown here for comparison with the Bayesian detection results.

## 3. Overview of the Method

The observed atmosphere is considered to be in one of three states: clear, cloudy, or ashy. Numerical weather prediction (NWP) fields are used to forward model (simulate) observations of each of the states by means of a radiative transfer model (RTM). The simulations are convolved with uncertainties to generate probability density functions (PDFs) in observation space for each of these states, P(**y**|**x**,*c_i_*). The conditional probability of an observation (**y**) being made for a scene in each of the three states (*c_i_*), given time- and space-specific prior information (**x**) for the pixel, is then the PDF P(**y**|**x**,*c_i_*). The prior information that constitutes **x** may vary according to what information is available for the imaged scene and what information is accepted by the model used to calculate P(**y**|**x**,*c_i_*) from **x** and *c_i_*. The elements that make up the observation vector **y** are typically the brightness temperatures (BTs) recorded at wavelengths corresponding to the spectral channels of the sensor that acquired the imagery. The conditional probabilities P(**y**|**x**,*c_i_*) are combined with prior probabilities P(*c_i_*) for each state using Bayes theorem to calculate the posterior probability that a pixel observation corresponds to each of the three states, equation 1.

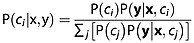
1

This is the equation from which the two-state classification problem for cloud detection was derived in *Merchant et al*. [2005]. The following sections present an implementation of this method for volcanic ash detection, providing an example of how the terms in equation 1 may be derived.

### 3.1. PDF for Clear Sky, P(**y**|**x,*c*_clear_)**

For the clear-sky case, we use NWP fields (with all cloud fields removed) to drive the fast RTM, RTTOV v.10 [*Saunders et al*., 1999, 2013], to simulate the anticipated BTs for a clear-sky observation made at the pixel location. The simulated observation is convolved with assumed Gaussian uncertainties to create a distribution in observation space. This distribution is the PDF for clear sky from which P(y|x,*c*_clear_) can be read.

Following *Merchant et al*. [2005] and *Mackie et al*. [2010a, 2010b], it is assumed that the uncertainties inherent to the simulations are dominated by the forward modeling sensitivity to surface temperature (ST), total column water vapor (TCWV), and, for land pixels, surface emissivity (SE), and that these sensitivities are independent. The RTM sensitivity to these terms is found by repeating the RTM run with a small perturbation to each of these parameters in turn, all other NWP fields remaining unchanged. This generates the **H** matrix, shown in equation 2 where *n* is the number of spectral channels used for the classification. The assumption of independence for the sensitivities is necessary for the implementation of equation 2 in equation 5 later, although some interdependence is likely in reality.

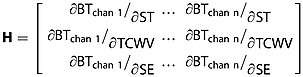
2

The uncertainty ***σ*** attributable to each of these NWP fields is also assumed independent, although as for the **H** matrix, this is unlikely to be the case in reality and is a computational necessity rather than ideal. The uncertainties are held in matrix **B**, equation 3.

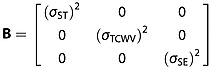
3

The signal-to-noise ratio for the sensor (NEdT) is held in the **R** matrix for each spectral channel, combined with the absolute uncertainty attributable to the forward modeling (FM) as shown in equation 4.

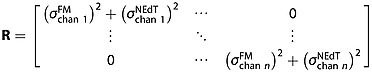
4

As with **H** and **B**, the assumption of a diagonal matrix for **R** is necessary to make the problem computationally practical and is made following *Merchant et al*. [2005] and *Mackie et al*. [2010a, 2010b], but it is possible that in reality the forward modeling errors have some interchannel dependency. These uncertainty terms are used to “spread” each simulated observation into a distribution in the observation space (the PDF for clear sky) following the convolution in equation 5, in which **y^o^** and **y^b^** are the observed and modeled BTs, respectively [*Merchant et al*., 2005]. Equation 5 can be computationally expensive and becomes impractical to implement on a standard desktop machine if **y** has more than three elements (i.e., if more than three spectral channels are used), although this may change as computer power grows. The assumption of diagonal matrices for **H**, **B**, and **R** and the assumption that all uncertainty in the NWP inputs to the forward model is accounted by the uncertainties attributable to ST, TCWV, and SE mean that the resulting PDF may be narrower than is appropriate. This is partly addressed through making conservative estimates for the terms in **R**, **B**, and **H**, and previous works have shown that the resulting characterization of clear (and cloud) observations is not too narrow to produce convincing results [*Merchant et al*., 2005; *Mackie et al*., 2010a, 2010b].

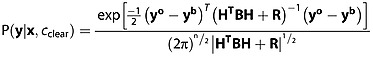
5

#### 3.1.1. A Priori Data and Uncertainties for the Clear-Sky PDF

NWP profiles on 91 vertical model levels were taken from the European Centre for Medium-Range Weather Forecasts (ECMWF) short range forecast model, IFS (http://www.ecmwf.int/research/ifsdocs/), for the image acquisition time and used for the a priori information (**x** in equation 1) for this demonstration of the technique. The uncertainty in ST required for **B** in equation 3 is taken from *Mackie et al*. [2010a], where a conservative estimate was used and is shown in Table  1. Following that same work, the uncertainty in TCWV is conservatively estimated as 21%. The uncertainty in the emissivity is provided with the emissivity atlas utilized for this demonstration and varies with season and latitude [*Borbas and Ruston*, 2010]. The values for **H** are calculated for each clear-sky simulation by running the same simulation with slightly perturbed input parameters and so are specific to each pixel. The sensor signal-to-noise ratio, NEdT, is read from *Hilton et al*. [2011] as 0.27 K for 10.65 µm and 12.00 µm, and 0.5 K for 3.9 *μ*m. This is combined with RTM uncertainties taken from *Saunders et al*. [2011] to calculate the terms in **R** in equation 4.

**Table 1:** Uncertainties Attributable to the NWP ST Field

	*σ*_ST_ (K)
Sea Day	0.88
Sea Night	0.88
Land Day	2.4
Land Night	1.6

It should be noted that in all cases, conservative estimates were made for the uncertainties where exact numbers were not available. This is appropriate because there are likely to be further uncertainties inherent in the technique that are not explicitly captured, for example, those attributable to NWP fields other than ST, TCWV, and SE.

### 3.2. PDF for Cloud, P(**y**|**x,*c*_cloud_)**

Calculation of the PDF for cloud is more complex than for clear sky since it must represent many atmospheric states, corresponding to clouds at different altitudes, with different thicknesses and different water/ice compositions. To make the method computationally practical, these calculations are done offline and NWP-dependent look-up tables (LUTs) produced. These LUTs are used in the same way as the clear-sky PDF, that is, NWP data for the observation determine which LUT it is appropriate to use, and the observation provides the coordinates for P(**y**|**x**,*c*_cloud_) in the LUT for that pixel.

We use a data set of 10,000 atmospheric profiles from the ECMWF IFS model to create the LUTs. The profiles are sampled to be as representative as possible of spatial and temporal variations in atmospheric temperature and water vapor [*Chevallier*, 2006]. It is important that the variability of these two parameters be realistically represented in the PDF for cloud (and for ash) because apart from the cloud itself, these are the atmospheric parameters to which IR observations are anticipated to be most sensitive (see section 3.1.1).

**Figure 1:**
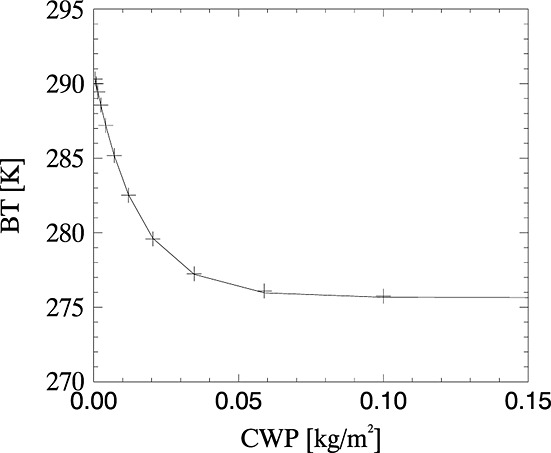
Curve fitted to simulated BTs at 10 µm for a water cloud at 4270 m altitude with 100% pixel coverage.

We follow the method presented in *Mackie et al*. [2007] and *Mackie* [2009] to add water and ice clouds to each atmospheric profile at specific model altitudes, with specific cloud water/ice path (CWP) values, and for different fractions of pixel coverage. Multiple observations of cloud are then simulated from each profile, each observation corresponding to a different combination of these parameters. Treating each profile individually, curves are fitted relating the simulated BTs to CWP. Each curve is specific to a single cloud phase (ice/water), altitude, and fraction, and is described by the expression BT = *a* + b*(1 − exp^−CWP/c^) where *a*, *b*, and *c* are parameters determined through iteration; see Figure 1. Interpolation of *a*, *b*, and c generates BT-CWP curves for clouds of the same phase at other altitudes and with different pixel fractions. BTs are read from the interpolated curves for each profile for clouds at 10 m vertical intervals through the atmosphere, with pixel fractions varying from 10% to 100% in 1% increments and with CWP increasing from 0.0005 kg/m^2^ to 0.5 kg/m^2^ in 99 exponentially spaced increments. For each profile, water and ice phase clouds are treated separately, as are the different spectral channels. These BTs, inferred from the simulated BTs, form the basis of the PDFs for cloud.

**Figure 2:**
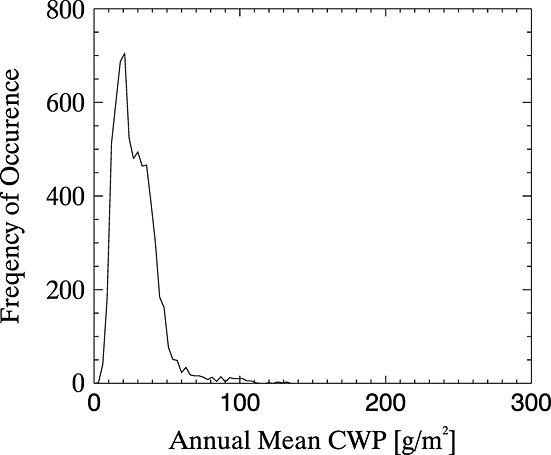
Frequency of occurrence of cloud water path (CWP) values in ISCCP mean annual water path data.

The maximum simulated CWP (0.5 kg/m^2^) ensures that clouds are represented up to optical saturation at all layers in the atmosphere, and the minimum reflects what, for the purposes of this work, constitutes “cloud,” meaning that clouds with CWP < 5 g/m^2^ are unlikely to be identified as cloud by this technique. The exponential CWP spacing can be justified by considering the frequency distribution of the global mean annual water path data available from the International Satellite Cloud Climatology Project (ISCCP) at (http://isccp.giss.nasa.gov) in Figure 2 [*Rossow et al*., 1996]. Optically thin clouds are likely to be unrepresented in these ISCCP data because cloud detection algorithms often miss these. Furthermore, both very thick and very thin clouds are unlikely to appear in the data set since it is composed of average values, hence the range of the ISCCP data being narrower than the range that it is appropriate to represent in the PDF. The shape of the density function, however, does serve to justify the exponential spacing of the represented CWP values.

**Figure 3:**
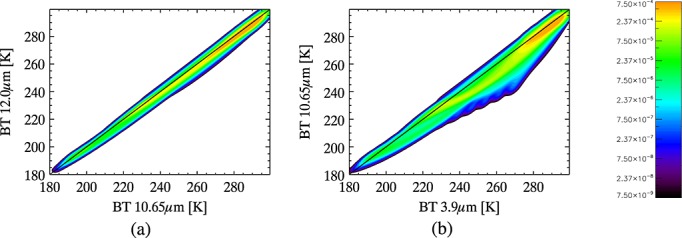
Example of the PDF for cloud for one ST-TCWV group (TCWV > 10 kg/m^2^, 280 K < ST < 300 K), for daytime observations made over sea.

**Figure 4:**
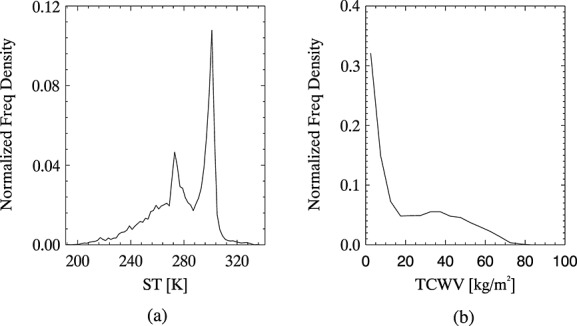
Frequency distribution for (a) ST and (b) TCWV in the ECMWF data set used to calculate the PDFs for cloud and ash.

The inferred cloud observations for each profile are weighted according to the relative likelihood assumed for each of the simulated clouds, given the atmospheric profile. The distribution of cloud observations for each individual profile is convolved with uncertainties using equation 5, as for the clear-sky case, creating a PDF for cloud specific to the atmospheric profile. The weightings are discussed in section 3.2.1, and the uncertainties are discussed in section 3.2.2. Since the profiles are assumed to be representative of all spatial and temporal variations in the noncloud parameters to which the observations are most sensitive, the profile-specific PDFs could all be averaged to create a PDF for cloud that is independent of the atmospheric data. This would be equivalent to setting P(**y**|*c*_cloud_) = P(**y**|x,*c*_cloud_) in equation 1. Some atmospheric conditions make certain types of cloud more likely than others, and it is preferable that our definition of a cloud class reflects a distribution of cloud properties that is realistic for the atmosphere above the pixel rather than being representative of all atmospheric conditions. To achieve this, profiles in the data set are grouped according to ST and TCWV and the profile-specific PDFs corresponding to each group are averaged to create group-specific PDFs for cloud, which are used as LUTs for the detection. For a given pixel, the appropriate LUT from which to read P(**y**|**x**,*c*_cloud_) is selected according to the ST and TCWV attributed to the pixel. An example is shown in Figure 3. Previous studies have noted that these two parameters affect the IR spectral signature for ash and can therefore affect the performance of more traditional detection methods, necessitating various correction techniques, e.g., *Yu et al*. [2002], and so using these to characterize an atmosphere for the purposes of ash detection is appropriate. Frequency distributions for ST and TCWV within the profiles data set were used to decide appropriate groupings; see Figure 4 and Table  2.

**Table 2:** Number of Atmospheric Profiles in Each ST-TCWV Group

Group	Day	Night	Total

*Sea Scenes*			
TCWV < 10 kg m^2^	961	1622	2583
ST < 280 K			
TCWV > 10 kg m^2^	183	180	363
ST < 280 K			
TCWV < 10 kg m^2^	133	169	302
280 K < ST < 300 K			
TCWV > 10 kg m^2^	749	1265	2014
280 K < ST < 300 K			
ST > 300 K	538	1030	1568

*Land Scenes*			
TCWV < 30 kg m^2^	391	1102	1493
ST < 270 K			
TCWV < 30 kg m^2^	621	288	909
ST > 270 K			
TCWV > 30 kg m^2^	485	283	768

#### 3.2.1. Clouds Represented in the PDFs for Cloud

All clouds represented in the LUTs are both single phase and single layer; however, it is anticipated that multilayer and mixed phase clouds would correspond to observations in an area of the observation space already populated by the simpler clouds which are explicitly represented and would be unlikely to significantly alter the PDF shapes [*Mackie*, 2009]. It was therefore deemed unnecessary to explicitly model more complex clouds.

The RTTOV model used for the clear-sky simulations was also used for the cloud simulations. RTTOV can simulate six cloud types—stratus continental, stratus maritime, cumulus continental clean, cumulus continental polluted, cumulus maritime, and cirrus. All stratus and cumulus cloud types are modeled as water clouds, with spherical water droplets and a gamma size distribution [*Matricardi*, 2005]. Cirrus clouds are conceptualized as hexagonal ice crystals, with scattering calculated following geometric optics for large crystals [*Takano and Liou*, 1989] and T-matrix calculations for small crystals [*Mishchenko et al*., 2000]. The assumed particle size distribution (PSD) for cirrus clouds is taken from *Heymsfield and Platt* [1984]. Since it is possible to calculate temperature-dependent weightings for the relative likelihood of water and ice phase clouds [*Mackie et al*., 2007], but not straightforward to judge the relative likelihood of more distinct cloud types, it is useful to simulate observations for a single type of water cloud and a single type of ice cloud. All water clouds were therefore simulated as cumulus clouds, and all ice clouds were simulated as cirrus, with the appropriate marine/continental cloud type selected according to the profile location.

To prevent unrealistic clouds being represented, some conditions were imposed to remove some simulated observations from the distribution and to weight the representation of some clouds more heavily than others.

**Figure 5:**
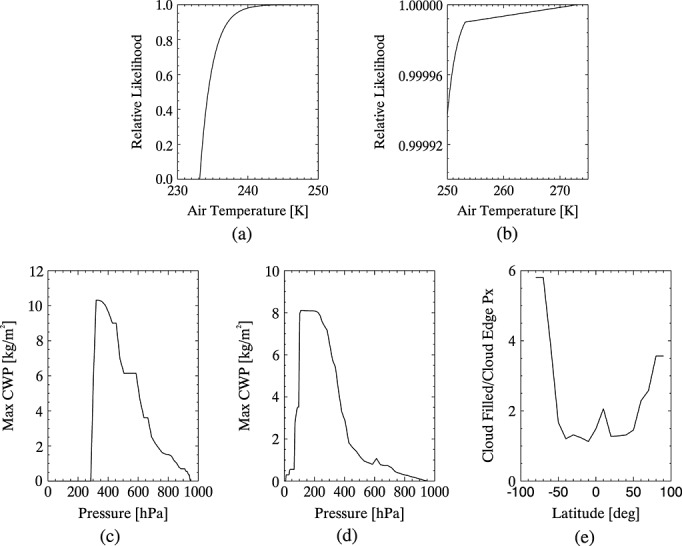
Restrictions and weights imposed on the representation of clouds within the PDF: (a, b) Temperature-dependent weighting applied to the representation of water clouds relative to ice clouds (the axes are scaled differently in Figures 5a and 5b to show the weighting functions applied in different temperature regimes), (c) the maximum cloud water path represented in the PDF as a function of cloud altitude for water clouds, (d) same as Figure 5c but for ice clouds, and (e) the ratio of cloud-filled to cloud-edge pixels in the ECMWF data set, used to weight the representation of clouds with 100% pixel coverage more heavily than those with fractional coverage.

Observations corresponding to ice phase clouds were removed from the distribution where the ambient temperature, *T*, exceeds 273.15 K and water phase clouds are removed where *T* is less than 233.15 K (at which temperature homogeneous freezing of water drops occurs [*Rogers and Yau*, 1989]). The likelihood of ice clouds forming where *T* > 233.15 K depends on the abundance of freezing nuclei, which are generally active when *T* < ~ 264.15 K, and deposition nuclei, which are generally become active around *T* ~ 253.15 K [*Rogers and Yau*, 1989; *Moran and Morgan*, 1997]. Ice clouds may therefore be present around *T* ~ 264 K but become more likely at *T* ~ 253 K, when both freezing and deposition nuclei are available. A relationship between the likely presence of ice nuclei and *T* is used to weight the representation of ice clouds, relative to that of water clouds, at each cloud altitude (i.e., for each value of *T*). A linear weighting was assigned to ice clouds where 273.15 K > *T* > 253.15 K and an exponential weighting where *T* < 253.15 K, reaching a value of 1 at *T* = 233.15 K. This follows the relationship between *T* and ice nuclei abundance presented by *Fletcher* [1962]. Supercooled water clouds can exist anywhere where *T* > 233.15 K, depending on the number of ice nuclei present [*Rogers and Yau*, 1989]. Therefore, water clouds are assigned a weight of one minus the Temperature-dependent ice cloud weighting where 273.15 K > *T* > 233.15 K. These weightings are summarized in Figure 5.

The maximum CWP for clouds at each altitude in the profiles data set was used as a cutoff, and no clouds with CWP greater than this (at the corresponding altitude) were represented. Since all clouds are represented as inhabiting a single model layer, the maximum CWP taken from the data set is the maximum integral of the CWP for every model layer up to and including the model layer at that altitude. An altitude-specific weight for the representation of optically saturated clouds is taken from the ratio of the number of ECMWF profiles containing cloud with CWP greater than the maximum simulated CWP to the number of clouds with CWP less than or equal to this. These weightings are shown in Figure 5. The use of statistics from the same data set of profiles as was used to calculate the PDFs is justified because all cloud fields were removed from the profiles prior to the RTM calculations.

Clouds are only expected to be present in saturated air masses (or near saturated if sea salt is present). The specific humidity at each simulated cloud altitude is used to calculate the maximum fraction of an NWP grid cell that could contain 100% relative humidity (70% for altitudes < 2 km over sea, since aerosol such as sea salt considerably enhances cloud production [*Junge et al*., 1969]). In cases where this value is greater than 1, it is raining. This altitude-specific maximum grid cell fraction is used to assign a relative weight to the representation of clouds at different altitudes for each individual profile.

Cloud-filled pixels are more likely than cloud-edge pixels, but in the simulated and interpolated clouds, the latter are more frequent. Cloud fraction data from the profiles data set were used to calculate the relative frequency with which full cloud coverage occurs at different latitudes. This was adjusted for the difference in spatial resolution between a pixel and an NWP grid cell and used to assign a latitude-dependent weight to the representation of full clouds relative to cloud edges; see Figure 5.

#### 3.2.2. Uncertainties in the PDFs for Cloud

Equation 5 is used to convolve the weighted distribution of cloud observations for each profile with uncertainties. The values in **B** and the sensor noise terms in **R** are those given in section 3.1.1 for the clear-sky case, since the calculations are based on atmospheric profiles from ECMWF's IFS model in both cases. For each observation represented in the PDFs for cloud, the latitude-dependent errors associated with forward modeling of cloud observations are taken from *Matricardi* [2005] and summed in quadrature with the clear-sky forward modeling error and the assumed NEdT to give the FM terms in **R** for cloud. A clear-sky simulation is carried out for each profile contributing to the cloudy LUTs and repeated with perturbed input parameters to calculate profile-specific RTM sensitivity to ST, TCWV, and SE for **H**. While it is unlikely that the clear-sky sensitivity of observations to these parameters equals the sensitivity of the simulated cloudy sky observations, this assumption is computationally practical and results in a conservative estimate of the uncertainties.

### 3.3. PDF for Ash, P(**y|x,*c*_ash_)**

An approach analogous to section 3.2 was initially attempted to create a NWP-dependent PDF for ash from modeled ash observations. This entails modeling all observations of ash that are theoretically possible for each atmospheric profile and combining them into profile-specific distributions, in which each observation is weighted according to the relative likelihood of the ash that it corresponds to being present in the profile. Following this approach, no particular ash characteristics should be assumed; instead, all possible ash characteristics are represented, making the PDF applicable to ash from any eruption. While it is possible to estimate the relative frequency with which different meteorological cloud properties occur, it is challenging to do this for ash since “ash” can have a range of properties and there are very few data available on the relative frequency with which they each occur. In the absence of such data (to weight the relative contributions of different ash observations to a PDF constructed in this way), all ash observations must be represented equally, creating a broad and flat PDF. If data from which to construct a realistic frequency distribution of ash characteristics did exist, it is possible that the relative frequency for a wide range of ash properties could be fairly similar, and a broad, flat PDF would again result. The Bayesian approach is most effective when the PDFs assumed for all the classes are well defined, meaning that observations are associated with very high (or very low) values and so can be strongly (or weakly) associated with each class. When the PDF for one class is broad (relative to the other classes), pixels are likely to be associated with that class as a result of not being strongly associated with the other classes rather than because of a high positive association. This will generally lead to more ambiguous classifications, and so the PDF for ash calculated in this way was not judged appropriate for implementation of the Bayesian technique. The poorly constrained range of physical properties encompassed by the term “ash” is a common problem in ash detection, where ash properties are often unknown. In the case of a well-monitored eruption, however, it is sometimes possible to use ground- and/or air-based observations to make a reasonable assumptions for the ash altitude, composition and PSD. We follow two approaches here and compare the resulting detection. In section 3.3.1, we construct a model-based PDF, in a similar way to section 3.2, but constraining the ash cloud to altitudes of 5–10 km and assuming its composition to be andesitic. These assumptions follow from observations of this specific ash plume [*Arason et al*., 2011]. The second approach, described in section 3.3.2, calculates an empirically based PDF from expert manual assessment of imagery from a range of eruptions (not including Eyjafjallajökull) and atmospheric conditions, and is therefore not “plume specific.”

#### 3.3.1. A Model-Based PDF for Ash

To simulate observations of ash, the RTTOV model was modified slightly to consider the optical properties of volcanic ash. The optical properties for andesite are taken from *Pollack et al*. [1973] and used as the refractive indices for the ash to be represented in the PDF. A lognormal PSD was assumed for the simulated ash, since this shape appears to best describe PSDs observed for ash clouds from a range of eruptions [*Hobbs et al*., 1982, 1991], including Eyjafjallajökull [*Johnson et al*., 2012] and has been assumed for many remote-sensing studies of volcanic ash from this eruption [e.g., *Stohl et al*., 2011; *Millington et al*., 2012]. The assumed PSD considers only fine ash particles, since larger particles do not scatter light at IR wavelengths and therefore are unlikely to be detectable at IR wavelengths and also because in the far field it is reasonable to expect ash clouds to be dominated by finer particles. For example, *Bukowieck et al*. [2011] and *Johnson et al*. [2012] studied ash samples collected in situ by aircraft following the eruption of Eyjafjallajökull in 2010. These studies found peaks in the volume distribution for particle diameters around 3 and 4 µm respectively. The latter study found less than 10% of the mass to be accounted for by particles with diameters greater than 10 µm. Ash particles are known to be angular rather than spherical; however, the assumption of sphericity has been found to have an insignificant effect on radiances simulated at IR wavelengths [*Yang et al*., 2007], and we therefore assume spherical particles here. The PSD and refractive indices are used in Mie scattering calculations to obtain the absorption, scattering, and backscatter cross sections that are required by RTTOV for the simulation of volcanic ash observations.

**Figure 6:**
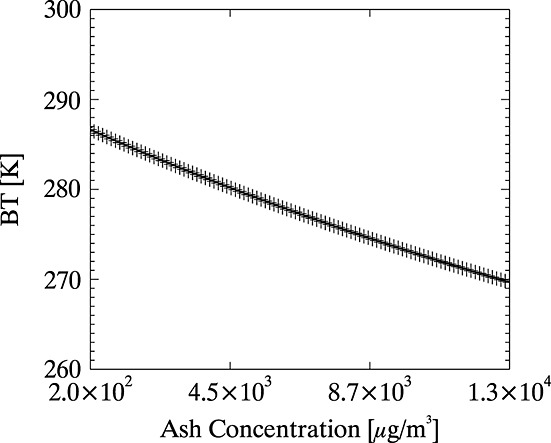
Curve (solid line) fitted to the simulated BTs at 10 µm (plus) for an ash cloud at 9755 m altitude (note that the *x* axis has a logarithmic scale).

We use the data set of atmospheric profiles from section 3.2 to forward model observations of ash. Multiple ash observations are simulated for each profile, corresponding to ash at different altitudes and with different concentrations. Curves are fitted to the simulated ash observations, assuming the same form for the BT relationship with ash concentration, as assumed for the BT-CWP in section 3.2; see Figure 6. Following the same interpolation as in section 3.2, BTs for ash clouds were inferred for ash clouds at 3 m vertical increments through the atmosphere, with mass concentration varying from 50 µg/m^3^ to 13,000 µg/m^3^ in 1000 equally spaced increments for each individual profile.

**Figure 7:**
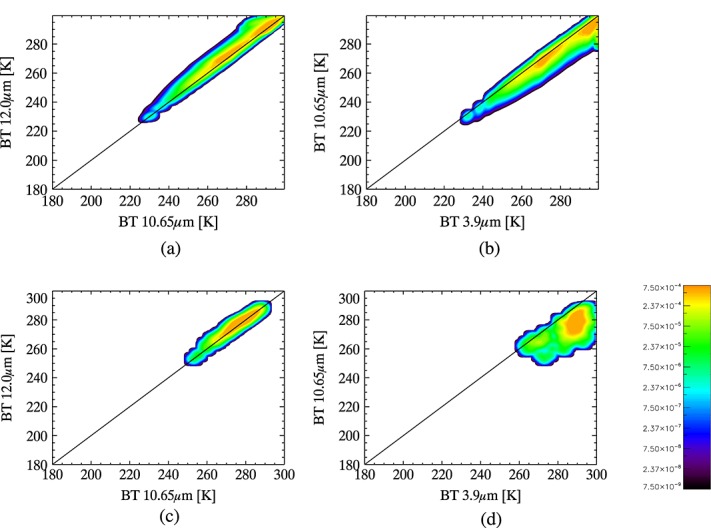
PDFs for ash for daytime scenes over sea: (a, b) using simulated observations and (c, d) using the empirical approach.

Each of these inferred ash observations contributes to a profile-specific distribution of observations, according to a weight that corresponds to the relative likelihood of the specific ash that it represents. These weights are discussed in section 3.3.1.1. This distribution of ash observations is convolved with uncertainties using equation 5, as for the clear and cloud cases but with added uncertainties for ash that are described in section 3.3.1.2, creating a profile-specific PDF for ash. As it is not possible to be confident that the distributions represent all possible ash states, and that the relative likelihood of the different states is weighted realistically, it is difficult to be confident that that the positioning of the PDFs in observation space is accurate. The effect of a slightly wrongly positioned PDF on the detection accuracy increases as the breadth of the PDF decreases and so, in contrast to the cloud case, NWP-dependent PDFs for ash are considered to be inappropriately narrow. Increasing the assumed uncertainties broadens the PDF but does not change its shape or position (since Gaussian uncertainties are assumed for each individual ash observation, the shape of the final PDF is determined by the relative weight assigned to the representation of the different ash states). We assume that by representing a large number of ash observations in the PDF, we overcome the problem of the range of possible states not being represented as appropriately as would be desirable and so achieve an appropriately shaped distribution; see Figure 7. This is equivalent to setting P(**y**|*c*_ash_) = P(**y**|x,*c*_ash_) in equation 1. Use of this PDF for all atmospheric conditions can be justified by the representativeness of the profiles data set, and while independence from NWP is not ideal, the classification retains its NWP dependence through the NWP dependence of the PDFs for clear and cloud.

##### 3.3.1.1. Ash Represented in the Model-Based PDF for Ash

Observations were explicitly modeled for ash in single model layers, for every model layer at between 5 and 10 km altitude, with 100 equally spaced mass concentrations ranging from 50 to 13,000 µg/m^3^, for every atmospheric profile. The lowest ash concentration considered hazardous to aviation is 200 µg/m^3^ [*Volcanic Ash Advisory Centre—London*, 2011]; however, it is appropriate to consider lower concentrations of ash for several reasons. This lower limit may change in future as more research is carried out into the effect of volcanic ash on aircraft, concentrations exceeding 50 µg/m^3^ can be considered harmful to health [*European Parliament and Council*, 2008], and the mass concentration may not be wholly accounted for by the fine ash to which the sensor is sensitive (i.e., 200 µg/m^3^ of ash may contain less than 200 µg/m^3^ of detectable fine ash). The upper limit corresponds the maximum ash concentration observed by a study of resuspended ash in Iceland [*Thorsteinsson et al*., 2012]. This concentration was measured close to the eruption source, and it is reasonable to expect mass concentrations to be lower in the far field because of sedimentation processes. However, it is not impossible for such a high concentration of fine ash to be encountered, it is just very unlikely, and it is therefore appropriate that high concentrations be represented, but that their representation is weighted realistically. Including high ash concentrations also goes some way to offsetting the single-layer cloud approximation.

**Figure 8:**
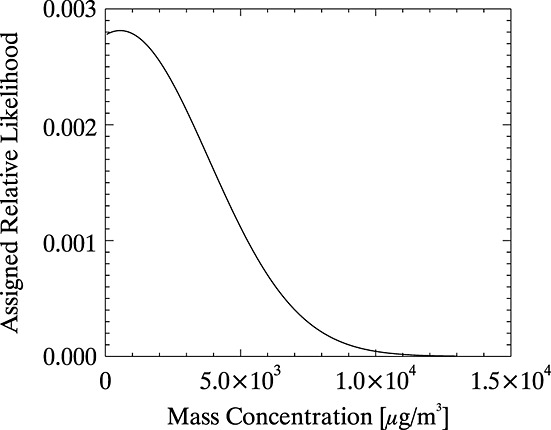
Relative weighting assigned to the representation of different ash mass concentrations in the model-based PDF for ash.

To ensure that the PDF for ash is as realistic as possible, the representation of different ash mass concentrations should be weighted according to what is known of their relative likelihood of occurrence. *Marenco et al*. [2011] looked at airborne lidar observations of volcanic ash from this eruption and fitted Gaussian distributions to the frequency with which different mass concentrations were observed. Four distributions are presented in that work, corresponding to four flight legs. These can be combined to give a single normalized Gaussian distribution representing the relative likelihood of occurrence of different mass concentrations on those flight legs. Flights could only take place where the forecasted ash concentration was lower than 2000 µg/m^3^ so this distribution is likely to be heavily biased toward lower concentrations (the presented data do, however, drop off toward higher concentrations within the measured range). The four selected flight legs were chosen in that work because they included encounters with reasonably high ash concentrations, which may mean that very low ash concentrations are also under represented. The Gaussian distribution was therefore smoothed several times by means of a Gaussian weighting kernel before being applied as a weight for the representation of different mass concentrations in the PDFs for ash, as shown in Figure 8. Ideally, this weighting function would be based on data measured in situ from all parts of the plume rather than just those parts safe for aircraft flight. In the absence of such a data set, however, these data form the most appropriate available basis for the weighting (the alternative being to weight all mass concentrations as equally likely or to use an arbitrary function).

It is unlikely that the calculated distribution of ash observations reflects the true distribution, since the prevalence of ash at all altitudes within the modeled range is unlikely to be uniform. Similarly, the relative likelihood for different ash concentrations is used to apply only a weak weighting (only a small deviation from a weighting in which all concentrations are considered equally likely); however, it is likely that the frequency with which ash occurs in the atmosphere varies more strongly with concentration than our representation allows for and a stronger weighting could be applied if there were data from which to calculate it. Furthermore, aggregation and sedimentation processes may mean that some ash states are more or less likely given the amount of water vapor present, and no weight is applied to the representation of different ash states in the PDF on this basis. For these reasons, it is appropriate to represent as many observations of ash as possible in the PDF and a global PDF, rather than narrower NWP-dependent LUTs, is constructed.

##### 3.3.1.2. Uncertainties in the Model-Based PDF for Ash

In addition to the sensor noise and clear-sky RTM uncertainties in section 3.1.1, the simulated ash observations are associated with an error from the implementation of the aerosol scattering scheme in RTTOV and with an uncertainty attributable to the assumed optical properties for the ash. The former is highly variable and has been shown to be higher for desert dust (likely to be similar to volcanic ash) than for other aerosols, but it remains below 1 K for all aerosols [*Matricardi*, 2005]. A value of 1 K is therefore conservatively assumed for this error. The second additional uncertainty is more difficult to assess—different compositions of volcanic ash correspond to different optical properties, and it is unlikely that the imaged ash is comprised of pure andesite with a lognormal PSD throughout the plume, as assumed for the RTM calculations. To assess this uncertainty, the ash cloud simulations carried out for the ashy PDF calculations were repeated on all profiles in the data set using RTTOV v.10's default volcanic ash optical properties (updated in RTTOV v.11). These are based on refractive index data calculated for “volcanic dust” in *Volz* [1973] and a gamma-shaped PSD. The difference between observations simulated under these assumptions and observations simulated under the assumptions described in 3.3.1.1 provides a measure of the sensitivity of the RTM to these choices and can be used to estimate the uncertainty attributable to them. Recent work has shown the range of optical properties that follows from different assumptions of refractive indices for ash [*Mackie et al*., 2014]. The data from Volz, which were measured from ash samples collected from Irazu Volcano, were demonstrated to lie at the edge of the range. By comparing against these data, rather than against another data set more similar to the andesite data set from Pollack, it is probable that we overestimate the uncertainty attributable to the choice of optical properties. As discussed in section 3.1, it is likely that uncertainties exist other than those that are explicitly accounted for, making an overestimation of the uncertainty attributable to any individual source preferable to the risk of underestimation. The difference between the two sets of simulated observations is likely to be dependent on the thickness of the ash cloud; however, equation 5 is implemented once per profile, convolving all the ash observations inferred for one atmospheric profile with the same uncertainties. The uncertainties are then profile specific but not specific to individual ash states. For each profile, the robust standard deviation was calculated from the distribution of the differences between the two sets of ash observations simulated for that profile and used as the profile-specific uncertainty attributable to the choice of optical properties. For each observation represented in the ash PDFs, these errors are added in quadrature to the clear-sky RTM error and the assumed sensor noise NEdT to calculate **R** for the ash case.

#### 3.3.2. An Empirically Based PDF for Ash

##### 3.3.2.1. Data for PDF for Ash

IASI images from the eruptions of Grimsvötn (2011), Kasatochi (2008), Nabro (2011), Puyehue (2011), and Sarychev (2009) are used to construct an empirically based PDF for ash. These eruptions produced ash clouds at altitudes where detection for aviation hazard monitoring is important and encompass a range of ash compositions—for example, ash from Puyehue was observed to have a very high silicate content [*Klüser et al*., 2013], while ash from Grimsvötn had a relatively low silicate content [*Marzano et al*., 2012]. Table 3 lists the acquisition times for the images, which were chosen to include both day and night scenes, and a range of latitudes. Ideally, statistics on the frequency with which different ash compositions and cloud heights occur and the relative frequency with which ash is present at different latitudes would be used to inform this sampling and make it more appropriate. Such statistics cannot be based on eruption data alone since these describe ash “injections” into the atmosphere, which must be combined with data on atmospheric residence times in order to form a data set that could be used to calculate these statistics. In the absence of these statistics, it is a necessary assumption for this work that the distribution of ash properties represented by the sampled ash observations is representative of all ash. There is some precedent for making such an assumption—*Clarisse et al*. [2010] successfully used spectra observed from the eruption of Eyjafjallajökull as characteristic ash spectra for detection of ash from some other eruptions. Also, ash detection and retrieval techniques using radiative transfer calculations generally assume a fixed composition for ash in order to model its optical properties [e.g., *Francis et al*., 2012], and this is unlikely to match the composition of all the ash for which the technique is applied.

**Table 3:** The Times Shown are the Acquisition Start Times for the Images in UTC

Eruption	Image Acquisition
Grimsvötn	23 May 2011 20:11:59
Grimsvötn	24 May 2011 09:47:59
Kasatochi	9 August 2008 20:53:55
Nabro	25 June 2011 17:06:00
Puyehue	7 July 2011 01:27:00
Puyehue	10 June 2011 00:23:55
Sarychev	16 June 2009 10:17:52

##### 3.3.2.2. Calculation of an Empirical PDF for Ash

Two independent experts, experienced in ash detection in thermal satellite imagery, examined the IASI images listed in Table 3 and selected those observations that they were confident contained ash. Only those observations identified by both experts as ash were used. The BT spectra for the selected observations were standardized by dividing by the BT at 8 µm and were grouped according to whether they corresponded to day or night, land, or sea. It was assumed that ash spectra in each of these groups from a single IASI image would be highly similar and some quality control was carried out on this basis. For each group, the mean standardized ash spectrum was calculated and the number of standardized spectra falling within three standard deviations of this was counted. If fewer than 95% of the spectra in the group fell within this range, then those outside this range were discarded, and the step was repeated until at least 95% of the spectra fell within three standard deviations of the mean spectrum for that image. The ash observations that passed this quality control were used to populate frequency distributions for day and night scenes imaged over land and sea, with a resolution of 0.5 K. To account for anticipated sensor noise and for the fact that only a limited number of image scenes were examined, these distributions were convolved with a Gaussian weighting kernel with a sigma value of 3 (corresponding to 1.5 K) to create the empirical PDFs for ash in Figure 7.

It is likely that one or both experts missed some ash in each image, and it is also likely that the similarity requirement for all ash spectra in a single image resulted in the exclusion of some ash observations. However, in the absence of an available data set of verified ash observations that covers a representative range of ash compositions and altitudes, this method was judged to be the most appropriate method of calculation for an empirical ash PDF.

### 3.4. Prior Probabilities

The prior probability for each state, P(*c_i_*), should be independent of any information used for the conditional probability for that state; i.e., it should not depend on the NWP fields. A prior probability for ash based on distance from the eruptive source in space and/or time was considered; however, it is difficult to conceive of a function that is independent of wind speed and direction. Furthermore, there may be cases where detection is desirable but where the eruptive source is unknown, e.g., for a new eruption of an unmonitored volcano. A fixed value is therefore assumed for P(*c*_ash_) and set somewhat arbitrarily at 5%. The difference between the conditional probabilities for the different states should be orders of magnitude greater than the difference between the prior probabilities, and so this assumption should only very weakly affect the posterior probabilities. Nonetheless, more data on which to base a realistic estimate of the appropriate value would be useful. The remaining 95% prior probability is split between clear and cloudy states according to monthly mean total cloud amount statistics available from ISCCP. The ISCCP data are provided on a 2.5° equal area grid. For computational efficiency, these were grouped into 10° latitude bands and four seasons, with land and sea considered separately. The average total cloud amount for each group was multiplied by 0.95 and used to populate look-up tables for P(*c*_cloud_), and P(*c*_clear_) was set to 0.95 − P(*c*_cloud_).

## 4. Results

**Figure 9:**
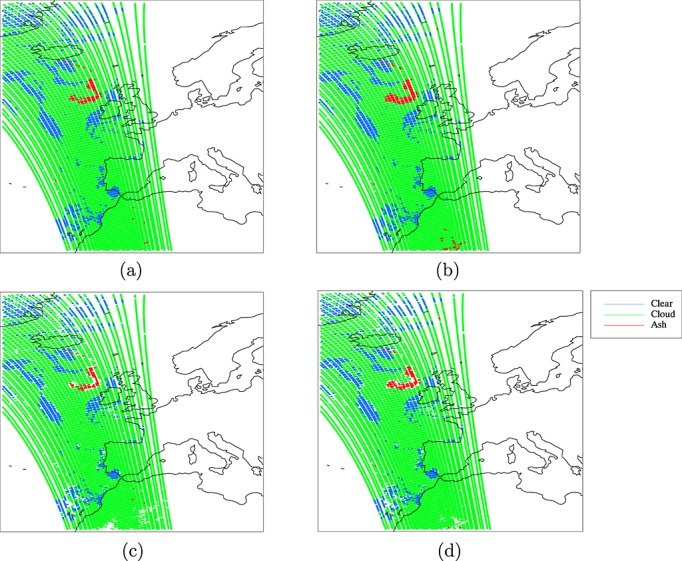
Classification for all pixels in the IASI image, using (a) the model-based PDF for ash and (b) the empirical PDF for ash. Classification for pixels in the IASI image with ambiguous classifications omitted, using (c) the model-based PDF for ash and (d) the empirical PDF for ash.

**Figure 11:**
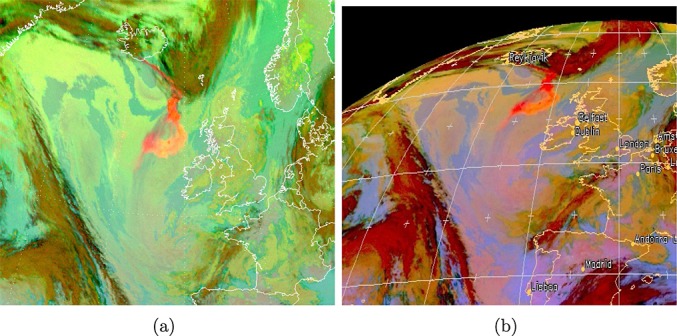
False color images from SEVIRI data for 6 May 2010: (a) “Dust” composite image for 21:00 image and (b) “Ash” composite image for 20:00 image. Ash appears pink in both composites. Images provided courtesy of EUMETSAT (http://oiswww.eumetsat.org/IPPS/html/MSG/RGB).

The calculations in section 3 were fed into equation 1 to calculate posterior probabilities for each of the three classes for each pixel and pixels were assigned to the class with the highest posterior probability. A further test was applied to identify ambiguously classed pixels. If the posterior probability for the assigned class was less than 0.6 or differed from the posterior probability for the next most likely class by less than 0.2, then the pixel was flagged as ambiguous. Two sets of results were generated—one using the model-based PDF for ash and one using the empirical PDF. The classified images are shown in Figure 9, both before and after removal of ambiguous pixels. The certainty associated with pixels classified as ash can be inferred from Figure 10, which shows the posterior probability for ash for all pixels, regardless of their final classification. Ideally, these results would be tested against a “test image” in which ash had been detected by some other means with complete accuracy. To the authors' knowledge, no such data exist and the most straightforward means to attempt to create them would be to use manual inspection of imagery by experts to classify individual pixels. It would be inappropriate to use expertise that is likely to contain similar biases to the expertise which was exploited for construction of the empirically based PDF for ash (since the “true” ash mask would be likely to contain the same biases as the results it was used to judge), and it is difficult to see how this could be prevented if such a “truth” were to be constructed. For comparison, therefore, we present the results alongside the false color composite imagery described in section 2, in which ash appears pink (Figure 11).

**Figure:**
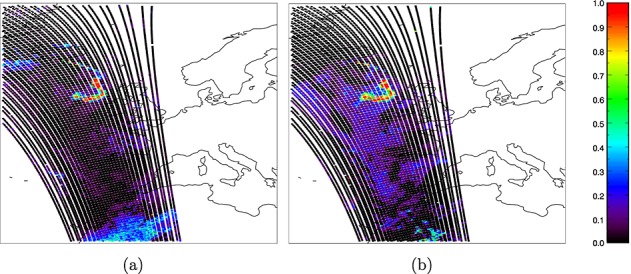
Posterior probability for ash calculated for all pixels, using (a) the model-based PDF for ash and (b) the empirical PDF for ash.

**Figure 12:**
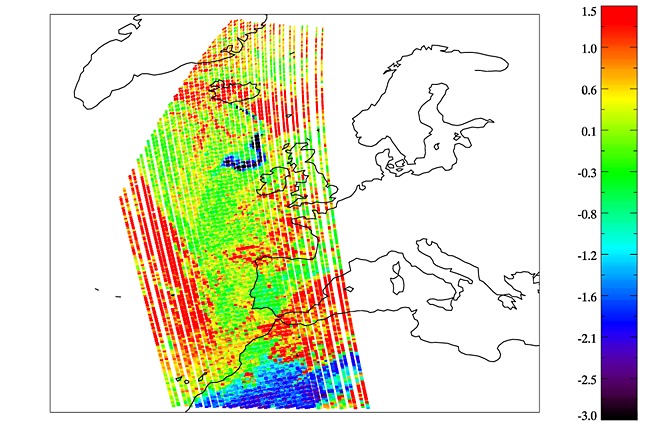
Brightness temperature difference (BTD) for the processed IASI image. The data are BT(10.65 µm)–BT(12.0 µm), shown in Kelvin.

For further comparison, the difference between BTs recorded at 10.65 µm and at 12.00 µm (BTD) is shown in Figure 12 since this is also a common indicator of ash. Thresholds could be applied to both the BTD and the false color products to produce binary masks for a more quantitative comparison; however, it is likely that any threshold would result in some ash being missed and some ash-free observations being falsely classified. Without thresholds, these products can be used to qualitatively evaluate the output from ash detection techniques such as that presented here and are therefore useful in an operational context when different products, which may not agree, are available.

**Figure 13:**
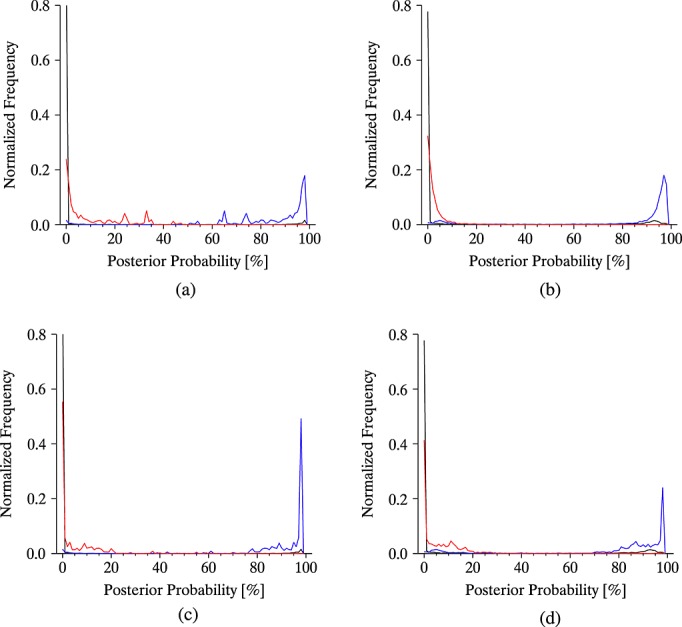
Frequency distributions for the posterior probability (%) calculated for each of the three states (clear, cloud, and ash). Using the model-based PDF for ash: (a) over land and (b) over sea. Using the empirical PDF for ash: (c) over land and (d) over sea. Clear is black, cloudy is blue, and ashy is red.

As a measure of the success of the technique, frequency distributions of the posterior probabilities for all three classes are shown in Figure 13, using the model and empirical PDFs for ash. If the method is effective, then the distribution of the posterior probabilities calculated for each class should be highly bimodal, indicating a high level of certainty to be associated with the overall classification.

## 5. Discussion of the PDFs for Ash

The success of the Bayesian approach is determined by the distinctiveness of the PDFs for the different states, and so the form of the PDF for ash is important. Ideally, a PDF in which all possible atmospheric states containing ash were represented would be used. A lack of validated ash observations from which to either construct a fully representative empirically based PDF or to constrain a model-based PDF means that such a generic PDF is impossible to achieve. A model-based PDF without appropriate constraints to reflect the relative likelihood of different ashy states would be too broad to be effective for the discrimination of ash. Ash clouds are highly variable in composition, shape, size, concentration, and altitude and so it may be that even with appropriate constraints, a generic PDF would be too broad to be useful for discrimination. The two approaches followed here both aim to create a narrow enough PDF for useful discrimination.

### 5.1. Empirical PDF for Ash

The empirical PDF cannot be fully representative since only a limited number of eruptions can be considered, and the number of ash observations from them is necessarily biased toward those that produced more ash and which were observed by the IASI satellite sensor. This is a problem since the properties of erupted ash are not known to always vary with the amount that is erupted or with the timing of satellite overpasses, and the selected eruptions are not known to be representative of all eruptions in time and space. Furthermore, the use of expert judgment to create the empirical PDF means that it is likely to only represent ash that would be detected by established techniques with which the experts are familiar, such as the BTD technique. This latter restriction is compounded by the quality control described in section 3.3.2.2. Although generally highly effective, the BTD method is known to be inappropriate for detection of ash in some atmospheric conditions, such as tropical atmospheres containing large amounts of water vapor [*Simpson et al*., 2000], and so the PDF is unlikely to explicitly represent such ashy atmospheric states. Implementation of the PDF in the Bayesian technique may still yield more effective discrimination than the BTD method alone since it is probabilistic (so uncertain classifications are identifiable), no thresholds are required, and it considers the three states simultaneously, meaning that an ash classification only requires an observation to look more like “ash” than like “cloud” or “clear,” where both “cloud” and “clear” are modeled according to the specific atmospheric conditions.

### 5.2. Model-Based PDF for Ash

Although the model-based PDF is calculated for the estimated properties of this specific ash plume, it is likely to be more generally representative of different atmospheric conditions than the empirical PDF. Since the variability of both the atmosphere and ash properties limits the scenes for which any single PDF is appropriate, it may be that the model-based PDF is also effective for discrimination of ash from other eruptions (although beyond the scope of this work, further research into this is likely to be useful). Conversely, the range of represented atmospheric conditions means that the PDF is likely to be broader and therefore may be less effective than desirable.

### 5.3. Comparison of the PDFs for Ash

Figure 7 shows that the two calculation methods result in quite differently shaped PDFs for ash. The model-based PDF extends to colder BTs, indicating that a wider range of temperatures is represented in the ECMWF profile data set than in the areas selected for construction of the empirical PDF. The two peaks in the model-based PDF correspond to the ST distribution in the profiles data set (Figure 4a) and so indicate the representation of thin ash clouds in the PDF. Since the data set is sampled to be representative of global variations in temperature and water vapor, these peaks can be assumed appropriate for a globally representative PDF. Since the model-based PDF is tuned to the ash for this particular plume, however, it may be more appropriate to limit the profiles used in construction of the PDF to those with ST values in the range expected for the imaged region at the time of image acquisition.

### 5.4. Discussion of Classification Results

In the absence of a “truth” against which to validate the classification, it is difficult to quantify the accuracy of either result; however, Figure 9 shows that both PDFs identify ash in agreement with the pink areas of the “ash” and “dust” color composite images produced by EUMETSAT (Figure 1). The channel combinations for these images are selected in order to identify dust and ash, and in both images, ash is associated with the color pink. There is a strong negative BTD signal over the Sahara and the North Sea for this scene (Figure 2). It is likely that the former corresponds to desert dust, and it is encouraging that very few of these pixels are flagged as ash by the Bayesian classifier in Figure 9. The model-based ash PDF results in only five likely misclassifications here, and this reduces to three when ambiguous classifications are removed. The empirical PDF results in more likely misclassifications in this area, probably because the empirical PDF is broader than the model PDF (so a greater range of observations will fall within it). However, its flatter shape means that observations that lie within it are likely to be associated with lower probabilities (since both PDFs must sum to unity), and this explains the reduction of likely misclassifications over the Sahara to three in Figure 9d. It is interesting that the three likely misclassifications in this area are not the same three pixels in both sets of results, suggesting that different “dust-like” ash properties may be represented in the two PDFs. Figure 2 shows the likely misclassifications resulting from the model PDF to lie separated from the main dust mass, and it is possible that this dust has different properties to the dust that makes up the larger continuous dust area to their south (it is even possible that these pixels contain ash from Etna, although this is impossible to test).

The negative BTD signal over the North Sea in Figure 2 is almost definitely ash and agrees with both the results from the Bayesian classifier and with the pink areas in Figure 1. The condition for ambiguous classification results in the removal of pixels around the plume edge, where the ash is likely to be thinner and less spectrally distinct. The empirical PDF for ash detects a wider plume than the model PDF. If the wider plume is correct (as Figures 1 and 2 suggest is likely), then it suggests that either too narrow a range of ash properties are represented in the model PDF or that the weighting of different ash properties is inappropriate, or both. In Figure 9, the plume appears discontinuous as detected by both PDFs, with the “gap” being filled by cloud. This is verified by Figures 1 and 2, which show that the gap is likely to be an area where the ash plume is obscured by overlying cloud. Some of the pixels in this region correspond to ambiguous classifications and so may be flagged for further investigation in a hazard situation rather than being forced into a cloud or an ash class (these pixels are likely to contain a mixture of ash and cloud).

Figure 10 shows that the posterior probability for ash is more bimodal when the model PDF is used, meaning that a more confident classification is achieved. This reflects the greater breadth of the empirical PDF that follows from its representation of a greater range of ashy states than were modeled. A broader PDF will generally result in more ambiguous classifications but may detect ash with a greater range of properties than a narrower PDF would identify. A narrow PDF will give fewer ambiguous classifications but may miss ash with properties other than those explicitly represented. Figure 3 shows that the posterior probability for each class is reasonably bimodal whichever of the PDFs is used, which demonstrates that the Bayesian technique can discriminate confidently, with few ambiguous pixels, for this scene. The higher uncertainties considered over land (which broaden the PDFs for all states) results in a higher number of ambiguous classifications over land than sea for all classes, and this is particularly marked when the model PDF is used for ash. The empirical PDF for ash results in slightly fewer ambiguous classifications over land but more ambiguous classifications over sea. In all cases, the distribution of probabilities for clear-classified pixels is more bimodal than for ash or cloud, indicating a higher degree of confidence for these classifications. This follows from the narrow shape of the PDF for clear sky, which is pixel specific and therefore does not represent as wide a range of atmospheric states. The bimodality of the distributions of calculated posterior probabilities for all classes shows that the method appears to perform well for the scene presented here using either PDF for ash.

## 6. Conclusions

The demonstrated technique is computationally efficient and produces a probabilistic product, thereby providing an inherent measure of the certainty for the classification of each individual pixel. In principle, it is not sensor specific and its exploitation of NWP data gives it a sound physical basis. The Bayesian method requires a PDF for ash, which can be calculated empirically or from model simulations. Examples of both approaches are used here and both perform well for this image. The model approach relies on appropriate assumptions being made for the properties of the ash, which may not always be possible. As more validated ash observations become available, it may be possible to calculate statistics with which to constrain a model PDF to be a tighter, narrower distribution that is fully representative of reality. If this becomes possible, then a model PDF is likely to perform better than an empirical PDF, which is unlikely to be representative of all ash in space and time. However, in the absence of such constraints, and prior knowledge about the imaged ash, an empirical PDF provides a practical solution and is demonstrated here to produce good results.
